# Bistatic Forward-Looking SAR Moving Target Detection Method Based on Joint Clutter Cancellation in Echo-Image Domain with Three Receiving Channels

**DOI:** 10.3390/s18113835

**Published:** 2018-11-08

**Authors:** Zhutian Liu, Zhongyu Li, Huaiqin Yu, Junjie Wu, Yulin Huang, Jianyu Yang

**Affiliations:** School of Information and Communication Engineering, University of Electronic Science and Technology of China, No. 2006 Xiyuan Ave, West Hi-Tech Zone, Chengdu 611731, China; huaiqin_yu@outlook.com (H.Y.); junjie_wu@uestc.edu.cn (J.W.); yulinhuang@uestc.edu.cn (Y.H.); jyyang@uestc.edu.cn (J.Y.)

**Keywords:** bistatic forward-looking SAR, moving target detection, keystone transform, joint clutter cancellation

## Abstract

In bistatic forward-looking synthetic aperture radar (BFSAR) ground moving target detection (GMTD), the suppression of the strong and heterogeneous ground clutter is one of the most crucial and challenging issues. Due to the bistatic forward-looking mode and long observation time, Doppler ambiguity, range and Doppler cells migration and non-stationary characteristics will exist in clutter receives, which leads to severe performance degradation of the traditional method. Hence, this paper proposes a GMTD method based on joint clutter cancellation in echo-image domain for BFSAR to achieve effective GMTD in heterogeneous BFSAR clutter. First, the pre-filtering and keystone transform are applied to suppress Doppler ambiguity and correct range cell migration, respectively. Then, time-division space-time adaptive clutter cancellation is adopted to suppress clutter at the first time in the echo domain, which can eliminate the effect of the migration of Doppler cells. However, its performance will be severely degraded due to the strong non-stationary characteristic of BFSAR clutter. Finally, adaptive displaced phase center antenna is exploited to suppress the residual non-stationary BFSAR clutter in image domain. Experimental results have shown that the strong non-stationary clutter of BFSAR has been sufficiently suppressed by the proposed method and the SCNR provided is enough to detect a moving target well.

## 1. Introduction

Synthetic aperture radar (SAR) is unique in disaster monitoring, resources exploration, military reconnaissance and security-related fields, due to its high-resolution day and night observation ability for areas of interest, independent of weather conditions. In general, a traditional SAR system is employed for stationary scene imaging [1,2,3]. However, in recent years, illegal activities, such as airport runway jamming and terrorism, have severely impacted everyday life and the safety of individuals. In order to prevent the occurrence of unexpected events mentioned above, the dynamic information real-time acquisition of the surveillance area has become a very important issue. As an additional function of SAR system, the ground moving target detection (GMTD) for SAR system has become increasingly attractive [4,5,6,7,8]. With the development of SAR technology, bistatic SAR system can break through the limitations of conventional monostatic SAR and achieve high-resolution imaging of the forward-looking terrain of the receiver. Therefore, bistatic forward-looking SAR (BFSAR) has received more and more attention in both civilian and military applications, due to its forward-looking observation ability and other advantages, such as high anti-interference, flexibilities and so on [9,10,11]. Thus, ground moving target detection for BFSAR has become an important research field [12,13,14,15,16,17].

In practice, when SAR system is working at downward-looking mode, receiver will generally receive massive strong ground clutter whose Doppler spectrum is broadened due to the platform motion. In this condition, ground moving targets are often overwhelmed by the strong clutter received, leading to a low signal to clutter and noise ratio (SCNR). As a consequence, clutter suppression is one of the crucial issues in SAR GMTD.

Current research and publications on SAR clutter suppression method are mainly divided into two types according to the system channel numbers: Single channel methods and multichannel methods. Clutter suppression methods based on single channel [18,19,20], such as Doppler filtering and interference processing, have a simple construction, but they usually cannot achieve a good performance due to the ground clutter Doppler spectrum widening. Multichannel methods mainly include a displaced phase center antenna (DPCA) [21,22,23,24,25] and space-time adaptive processing (STAP) [26,27,28,29,30,31]. The effectiveness of these multichannel methods has been verified in monostatic SAR systems. However, when it comes to bistatic SAR (BiSAR), ground clutter will present more complex characteristics, which will have a serious impact on STAP performance. The spectral distribution of clutter in BiSAR is closely related to the bistatic configuration, platform velocities and flight directions, while the Doppler frequency of BiSAR clutter is range-dependent [32,33,34]. As a result, there is a deviation between clutter plus noise covariance matrix (CCM) estimation and the true value of CCM, which leads to the severe deterioration of performance on STAP directly. Meanwhile, The Reed-Mallet-Brennan (RMB) rule indicates that in order to ensure that the output performance is corresponding to a 3 dB level below optimum, where I is the system degree of freedom (DOF), at least 2I + 1 independent and identically distributed (IID) secondary samples used in CCM estimation are required [35,36]. This data support is hard to satisfy and the computation is a burden for multichannel SAR systems. Hence, the ground clutter cannot be suppressed effectively by STAP with the wrong CCM estimation and the performance on GMTD will be affected greatly. On the other hand, the performance of DPCA will be strictly limited by its configuration limitation [21,23]. Contemporary, the equivalent phase centers of transmitting and receiving channels in BFSAR are not in the same location during a fixed time interval, because the transmitting channel and the receiving channel are mounted on different platforms. The phase error caused by the position deviation will vary with the change of azimuth time, which makes it not possible to get the precise phase compensation [24]. Therefore, the traditional DPCA method is also not usable for clutter suppression in BFSAR.

In addition, due to the forward-looking mode, the ground clutter of observed area will have a large radial velocity. And with the long observation time of BFSAR, three problems will be introduced into the received signal of BFSAR: Doppler ambiguity, range cell migration (RCM) and Doppler cell migration. The assumption that there is no range and Doppler migration during one coherent processing interval (CPI) will be invalid, which leads to the poor performance on traditional GMTD methods. In BFSAR, the azimuth Doppler ambiguity of the received signal will be caused by the large radial velocity of clutter. Range cell migration will lead to target energy dispersing over multiple range cells. Target signal will be aliasing with the superposition of the clutter from different range cells, which decreases the SCNR in detection. Also, the target signal cannot be directly distinguished from the clutter in the space-time domain due to its Doppler cell migration, which leads to severe clutter suppression performance deterioration. Consequently, the SCNR for GMTD in BFSAR cannot be improved enough from the raw SAR data by the traditional methods. Therefore, the traditional SAR GMTD methods are not suitable for BFSAR ground moving target detection.

This paper proposes a BFSAR moving target detection method based on joint clutter cancellation in echo-image domain with three receiving channels. The proposed method not only can eliminate the influence of Doppler ambiguity and range and Doppler cell migration on clutter suppression, but can also reduce the effect of non-stationary characteristic of BFSAR clutter and improve the SCNR for target detection. First, we use the pre-filtering and first-order keystone transform to suppress the Doppler ambiguity and correct range cell migration for the received signal, respectively. Then, time-division space-time adaptive clutter cancellation (STACC) is adopted between two adjacent receiving channels to suppress clutter at the first time in echo domain, which can eliminate the effect of the migration of Doppler cells. However, since the space-time distribution of BFSAR clutter is closely related to bistatic configurations and platform parameters, BFSAR clutter will present strong non-stationary peculiarity, which severely degrades the clutter suppression performance. As a result, massive non-stationary residual clutter will be retained and moving targets may be still buried by the residual clutter. Some compensation methods for the clutter non-stationary peculiarity, such as angle-Doppler compensation method and derivative-based updating method, are carried out. But these methods need the prior information of the angle and Doppler frequency of the clutter spectrum and high computational complexity, respectively. In order to suppress the strong non-stationary BFSAR clutter and lighten the computational burden of the system, adaptive DPCA (ADPCA) is exploited as the second clutter cancellation to suppress the residue clutter in image domain. With ADPCA processing, the clutter will be coarse focused and the difference of the phase between different channels can be accurately compensated in image domain. Thus, the residue non-stationary clutter of BFSAR will be sufficiently cancelled in the image domain. Experimental results of traditional STAP, direct time-division STACC after the correction of the cell migration and joint clutter cancellation in echo-image domain have shown that the strong and non-stationary ground clutter of BFSAR can be suppressed sufficiently by the proposed method and the SCNR can be improved greater, where a moving target can be well detected. 

The remainder of this paper is consisted by the following sections: Section 2 gives the signal model and echo characteristic analysis of BFSAR. Section 3 illustrates the proposed ground moving target detection method for BFSAR with three receiving channels, and the experimental results are shown in Section 4. The last section concludes this paper.

## 2. Signal Model and Echo Characteristic Analysis of BFSAR

**Figure 1:**
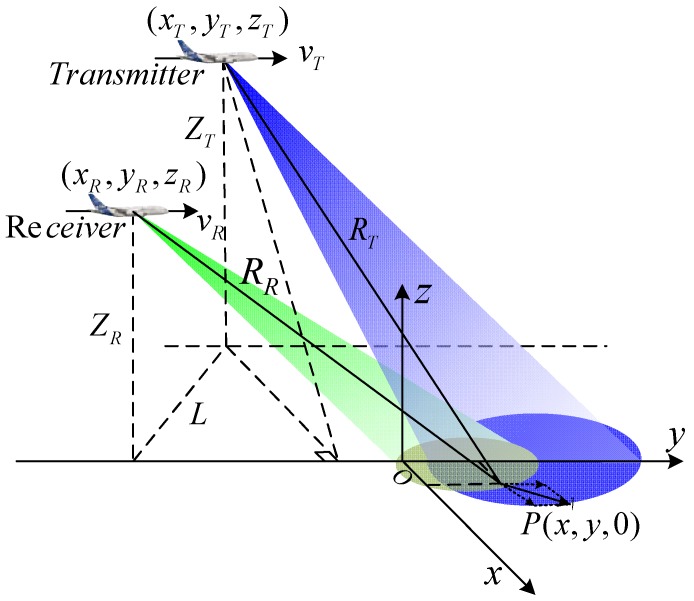
The geometry configuration of BFSAR.

The geometric configuration of bistatic forward-looking SAR is shown in Figure 1. The single channel transmitter and three channels equipped receiver are considered. The transmitter works in squint mode and the receiver works in forward-looking mode. The receiver and transmitter are both travelling along the y-axis with the velocity $v_{R}$ and $v_{T}$ respectively. The original coordinates of the receiver and transmitter are $\left(x_{R},y_{R},z_{R}\right)$ and $\left(x_{T},y_{T},z_{T}\right)$, respectively. L is the projection of the baseline on the ground. The channel spacing of receive channels is d. Assuming that the original coordinate of reference channel is $\left(x_{R},y_{R},z_{R}\right)$, the k-th channel is located at $(x_{R},y_{R}+(k-1)d,z_{R}),k=1,2,3$. The origin of the coordinate O is the scene center, and a moving target P with the velocity v is located at (x,y,0).

Supposing the transmitted signal is a linear frequency modulated (LFM) pulse, the received signal of the k-th (k=1,2,3) channel after demodulation can be expressed as

(1) $$\begin{array}{ll} S(t,\tau ,k;x,y)= & \omega_{r}\left(\tau -\frac{R(t,k;x,y)}{c}\right)\omega_{a}\left(\frac{t-t_{R}}{T_{s}}\right)\\ & \times \exp\left\{j\pi \left[K_{r}{\left(\tau -\frac{R(t,k;x,y)}{c}\right)}^{2}-\frac{2f_{c}R(t,k;x,y)}{c}\right]\right\} \end{array}$$

where $\omega_{r}$ and $\omega_{a}$ are the range and azimuth envelopes, respectively. τ is the fast time, t is the slow time and $K_{r}$ represents the range frequency modulated rate. $f_{c}$ is the carrier frequency and c is the speed of light. $T_{s}$ is the synthetic aperture time, and $t_{R}$ is the time the beam pattern center passing through the target. R(t,k;x,y) is the bistatic range history of the *k*-th channel, and it can be obtained by

(2) $$R(t,k;x,y)=R_{T}(t;x,y)+R_{R}(t,k;x,y),\;k=1,2,3$$

The range histories with respect to moving target P(x,y) of the receiver and the transmitter are

(3) $$R_{R}(t,k;x,y)=\sqrt{{\left(x_{R}-x-v_{x}t\right)}^{2}+{\left(y_{R}+(k-1)d-y+\left(v_{R}-v_{y}\right)t\right)}^{2}+z_{R}^{2}}$$

(4) $$R_{T}(t;x,y)=\sqrt{{\left(x_{T}-x-v_{x}t\right)}^{2}+{\left(y_{T}-y+\left(v_{T}-v_{y}\right)t\right)}^{2}+z_{T}^{2}}$$

where $v_{x}$ and $v_{y}$ are the cross-track and along-track velocity components of the moving target P, respectively. 

After range Fourier transform, the signal received by the *k*-th channel in range frequency domain azimuth time domain can be expressed by [37]

(5) $$\begin{array}{ll} S(t,f,k;x,y)= & \omega_{r}\left(\frac{f}{K_{r}}+\frac{R(t,k;x,y)}{c}\right)\omega_{a}\left(\frac{t-t_{R}}{T_{s}}\right)\\ & \times \exp\left\{-j\pi \left[\frac{f^{2}}{K_{r}}+\frac{2(f+f_{c})}{c}R(t,k;x,y)\right]\right\} \end{array}$$

Expanding the history range R(t,k;x,y) at the beam center crossing time $t_{R}$ into Taylor series, we have

(6) $$R(t,k;x,y)=R(t_{R},k;x,y)+R^{\prime }(t_{R},k;x,y)\left(t-t_{R}\right)+\frac{1}{2}R^{\prime \prime }(t_{R},k;x,y){\left(t-t_{R}\right)}^{2}+\cdots$$

where $R(t_{R},k;x,y)$ is the bistatic range sum of the transmitting channel and the *k*-th receiving channel at $t_{R}$, $R^{\prime }(t,k;x,y)$ and $R^{\prime \prime }(t,k;x,y)$ are the first-order and second-order derivatives of R(t,k;x,y), respectively. By setting $t_{R}=0$, the expanding coefficients R(0,k;x,y), $R^{\prime }(0,k;x,y)$ and $R^{\prime \prime }(0,k;x,y)$ are given by

(7) $$\begin{array}{l} R(0,k;x,y)=R_{0R}+R_{0T}=R_{R}(0,k;x,y)+R_{T}(0;x,y)\\ R^{\prime }(0,k;x,y)=\left[\left(v_{rad,R}(k)-v_{R}\cos\theta_{R}\right)+\left(v_{rad,T}-v_{T}\cos\theta_{T}\right)\right]\\ \begin{array}{ll} R^{\prime \prime }(0,k;x,y)= & \frac{v_{x}^{2}+{\left(v_{T}-v_{y}\right)}^{2}}{2R_{0T}}-\frac{{\left(v_{x}(x-x_{T})+(v_{y}-v_{T})(y-y_{T})\right)}^{2}}{2R_{0T}^{3}}\\ & +\frac{v_{x}^{2}+{\left(v_{R}-v_{y}\right)}^{2}}{2R_{0R}}-\frac{{\left(v_{x}(x-x_{R})+(v_{y}-v_{R})(y-y_{R}-(k-1)d)\right)}^{2}}{2R_{0R}^{3}} \end{array}\\ v_{rad,T}=\frac{v_{x}\left(x-x_{T}\right)+v_{y}\left(y-y_{T}\right)}{R_{0T}}\\ v_{rad,R}(k)=\frac{v_{x}\left(x-x_{R}\right)+v_{y}\left(y-y_{R}-(k-1)d\right)}{R_{0R}} \end{array}$$

where $\theta_{R}$ and $\theta_{T}$ are the downward-looking angle of the receiver and the squint angle of the transmitter, respectively. 

In BFSAR, due to the forward-looking mode, the first-order expanding coefficient $R^{\prime }(t,k;x,y)$ is always larger than pulse repetition frequency of the BFSAR system. The large $R^{\prime }(t,k;x,y)$ means that the Doppler centroid of clutter background and target signal is very large. As a consequence, the Doppler ambiguity in received signals will be caused by the large Doppler centroid, which will make range cell migration correction more difficult. Meanwhile, the large $R^{\prime }(t,k;x,y)$ leads to a severe coupling relationship between range and azimuth directions in signals. With the first-order coupling relationship, target energy will disperse over massive range cells than other bistatic configurations. The large-scale range cell migration will result in a tremendous decrease in the signal to clutter and noise ratio. Due to the long observation time of BFSAR, the Doppler frequency spectrum of one scattering point, which is depended on $R^{\prime }(t,k;x,y)$ and $R^{\prime \prime }(t,k;x,y)$, corresponds to a certain width during a coherent processing interval (CPI). Therefore, the assumption that there is no range and Doppler migration during a CPI is invalid, which causes major performance degradation of traditional GMTD methods [35,36].

## 3. Proposed Moving Target Detection Methods for BFSAR

Based on the previous analysis of BFSAR echo characteristics, a BFSAR ground moving target detection method based on joint clutter cancellation in echo-image domain with three receiving channels is introduced in this section. The proposed method mainly involves the following four steps. 

The bulk-deramp pre-filtering is applied as the first step to suppress the Doppler ambiguity, due to the Doppler ambiguity in BFSAR receives. Since the large-scale range cell migration of BFSAR can’t be corrected completely by the first-order keystone transform with the existence of Doppler ambiguity in the receives, the suppression of Doppler ambiguity is the important preparation for the following processing steps.

Then, the first-order keystone transform is applied as the second step to correct the large-scale range cell migration of BFSAR. After the keystone transforming operation, the range walk, which is the major component of the range cell migration in BFSAR, of the clutter background and moving target will be corrected completely. The energy of the moving target will be gathered into one range cell. However, the moving target will confront the superposition of the strong clutter at target range cell from different azimuth, which means that the signal of moving target is still overwhelmed by the clutter background after keystone transform. Therefore, clutter suppression is the crucial step for GMTD in BFSAR.

In order to suppress the strong ground clutter in BFSAR echo, twice clutter cancellation is exploited. Time-division space-time adaptive clutter cancellation is the third step to achieve the first clutter cancellation in data domain. Time-division STACC will be conducted between two adjacent channels of the system two times. The SCNR can be improved in echo domain at the first time. However, due to the non-stationary characteristic of bistatic clutter especially for the close distance situations, the CCM of BFSAR cannot be estimated accurately. As a result, the time-division STACC performance on clutter suppression will be severely degraded. Therefore, second clutter cancellation based on ADPCA in image domain is applied as the final step to suppress the residual non-stationary ground clutter after the cancellation in echo domain. After the sufficient joint clutter cancellation, the purpose of ground moving target detection can be easily achieved.

### 3.1. Doppler Ambiguity Suppression via Pre-Filtering

After the Tylor expansion of range history, the received signal of the k-th (k=1,2,3) receiving channel can be expressed as

(8) $$\begin{array}{ll} S(t,f,k;x,y)= & \omega_{r}\left(\frac{f}{K_{r}}+\frac{R(t,k;x,y)}{c}\right)\omega_{a}\left(\frac{t-t_{R}}{T_{s}}\right)\exp\left(-j\pi \frac{f^{2}}{K_{r}}\right)\\ & \times \exp\left\{-j2\pi \frac{\left(f+f_{c}\right)}{c}\left[\begin{array}{l} R(0,k;x,y)+R^{\prime }(0,k;x,y)t\\ +\frac{1}{2}R^{\prime \prime }(0,k;x,y)t^{2}+\cdots \end{array}\right]\right\} \end{array}$$

For the stationary background, due to the forward-looking mode of the receiver, the radial velocity of the scattering points in observed area is very large, namely the first-order coefficient $R^{\prime }(t,k;x,y)$ is very large. As a result, the severe coupling relationship between range and azimuth will be led into the received signal and the two-dimension spectrum of the scattering points will overlap to the adjacent PRF band. Therefore, the Doppler ambiguity exists.

In order to suppress the Doppler ambiguity of the BFSAR receive, the bulk-deramp pre-filtering function is constructed as

(9) $$H_{pre-filtering}=\exp\left(j2\pi \frac{(f+f_{c})f_{dc}}{f_{c}}t\right)$$

where $f_{dc}$ is the Doppler centroid of the reference point, and it can be given by

(10) $$f_{dc}=-\frac{{R^{\prime }}_{ref}(0,k;x,y)}{\lambda }=\frac{\left(v_{R}\cos\theta_{R,ref}+v_{T}\cos\theta_{T,ref}\right)}{\lambda }$$

where λ is wave length of the signal.

The signal without Doppler ambiguity can be obtained from multiplying the signal expression (8) by the filter function (10), we can have

(11) $$\begin{array}{ll} S_{filtered}(t,f,k;x,y)= & \omega_{r}\left(\frac{f}{K_{r}}+\frac{R(t,k;x,y)}{c}\right)\omega_{a}\left(\frac{t-t_{R}}{T_{s}}\right)\exp\left(-j\pi \frac{f^{2}}{K_{r}}\right)\\ & \times \exp\left\{-j2\pi \frac{\left(f+f_{c}\right)}{c}\left[\begin{array}{l} R(0,k;x,y)+(R^{\prime }(0,k;x,y)-\lambda f_{dc})t\\ +\frac{1}{2}R^{\prime \prime }(0,k;x,y)t^{2}+\cdots \end{array}\right]\right\} \end{array}$$

### 3.2. Linear Range Cell Migration Correctioin via First-Order Keystone Transfrom

From the Equations (6) and (7), we can know that the absolute values of $R^{\prime }(t,k;x,y)$ is much larger than that of $R^{\prime \prime }(t,k;x,y)$ and higher-order derivatives of R(t,k;x,y) and the range cell migration components caused by $R^{\prime }(t,k;x,y)$ is much larger than other components in BFSAR. Thus, the linear range cell migration is the major component of the range cell migration in BFSAR. 

After the bulk-deramp pre-filtering, the Doppler ambiguity caused by the large Doppler centroid has been suppressed and the coupling effect between range and azimuth directions has been weaken to some extent. However, we can find that the residual first-order coupling term is remained in the filtered signal $S_{filtered}(t,k;x,y)$. Thus, the target energy is still smearing over several range cells. In order to gather target energy in the one range cell, the residual first-order coupling term must be removed. The keystone is performed

(12) $$t=\frac{f_{c}}{f+f_{c}}t_{1}$$

where $t_{1}$ is the slow time variable after the keystone transform. The expansion of R(t,k;x,y) is keeping up to the second-order term, the phase term after keystone transform in Equation (11) has become

(13) $$\begin{array}{ll} \varphi (t_{1},f,k)= & -\frac{\pi f^{2}}{K_{r}}-\frac{2\pi (f+f_{c})}{c}R(0,k;x,y)\\ & -\frac{2\pi f_{c}}{c}\left(R^{\prime }(0,k;x,y)-\lambda f_{dc}\right)t_{1}-\frac{\pi R^{\prime \prime }(0,k;x,y)f_{c}^{2}}{c(f+f_{c})}t_{1}^{2} \end{array}$$

In Equation (13), the first-order polynomial of $t_{1}$ is $-2\pi f_{c}(R^{\prime }(0,k;x,y)-\lambda f_{dc})t_{1}/c$. The first-order coupling effect of range frequency f and slow time $t_{1}$ has been removed completely. Expanding the phase term into the Taylor series of range frequency and keeping up to second-order term, the phase in (13) can be expressed as

(14) $$\begin{array}{ll} \varphi_{f}(t_{1},f,k)= & -\frac{2\pi f_{c}}{c}\left(R(0,k;x,y)+(R^{\prime }(0,k;x,y)-\lambda f_{dc})t_{1}+\frac{R^{\prime \prime }(0,k;x,y)}{2}t_{1}^{2}\right)\\ & -\frac{2\pi }{c}\left(R(0,k;x,y)-\frac{R^{\prime \prime }(0,k;x,y)}{2}t_{1}^{2}\right)f-\pi \left(\frac{1}{K_{r}}+\frac{R^{\prime \prime }(0,k;x,y)}{cf_{c}}t_{1}^{2}\right)f^{2} \end{array}$$

The third term in Equation (14) represents the range modulation term, and the range compression can be carried out with the new range frequency modulated rate

(15) $$K_{new}=\frac{1}{\frac{1}{K_{r}}+\frac{R^{\prime \prime }(0,k;x,y)}{cf_{c}}t_{1}^{2}}$$

After the first-order keystone transform, the linear range migration has been completely corrected. Need to note that the quadratic range cell migration (the second term of the Equation (14)) is still retained. The linear range migration is more obvious compared with higher-order range cell migration. Thus, the effect of quadratic range cell migration can be neglected in BFSAR.

### 3.3. First Clutter Cancellation in Echo Domain

After the two steps presented above, the effect of Doppler ambiguity and the large-scale range cell migration have been eliminated. Although the energy of the target has been gathered into one range cell, the signal of the moving target is still submerged in the superposition of the strong clutter at target range cell from different azimuth. In order to improve the SCNR and detect the moving target from the strong clutter background, the two-channel time-division STACC is operated to achieve the first clutter cancellation in the SAR echo domain. 

For BFSAR, the Doppler spectrum of both ground moving target and clutter will span into multiple Doppler cell, because of their Doppler migration. Thus, the Doppler frequency of one scattering point corresponds to a certain range during a CPI, which makes the no Doppler migration assumption for traditional method invalid [35,36]. Under the circumstances, the frequency spectrum of the moving target and that of ground clutter are aliasing together in the Doppler domain. As a result, the moving target and the ground clutter may not be distinguished directly in the space-time domain, which vastly aggravates the difficulty of clutter suppression.

In order to eliminate the strong ground clutter of BFSAR, the effect of the Doppler cell migration must be removed. In this section, a clutter suppression method based on STAP for BFSAR is introduced to deal with the ground clutter with the Doppler range cell migration. 

For the long integration time of BFSAR, we can divide it into a huge quantity of periods, which are much shorter than a CPI. The length of one period can be decided by the azimuth resolution $\delta_{a}$ and frequency modulated rate $K_{a}$, and the length ΔL satisfied the inequation:

(16) $$\left\|\Delta L\cdot K_{a}\right\|\leq \delta_{a}$$

During each period, the Doppler frequency of the moving target and clutter scattering point can be approximated as an unchangeable constant. Thus, the signal processed by STACC in BFSAR can be regarded as a narrowband one in a period and the effect of Doppler cell migration is eliminated through the time division.

Then, the first clutter cancellation in SAR data domain can be carried out. For the range cell under detection, we can construct an optimal filter for space-time clutter cancellation in each period, the processing of space-time clutter cancellation in range time azimuth time domain is given by

(17) $$y\left(\eta \right)=W^{H}\left(\eta \right)x\left(\eta \right)$$

where η represents the processing period in azimuth time domain, W(η)∈2M×1 is the weight vector of the filter, x(⋅)∈2M×1 and y(⋅)∈1×1 are the input and the output of the filter, respectively. M is the number of pulses received during a period. According to the minimum mean square error (MMSE) rule, the problem of the optimal filtering can be described as a mathematical optimization model:

(18) $$\{\begin{array}{c} \min W^{H}\left(\eta \right)R\left(\eta \right)W\left(\eta \right)\\ s.t.\;W^{H}\left(\eta \right)s\left(\eta \right)=1 \end{array}$$

Then, the weight vector of the filter can be obtained by

(19) $$W(\eta )=\mu \left(\eta \right)R^{-1}\left(\eta \right)s\left(\eta \right)$$

where

(20) $$\begin{array}{l} \mu \left(\eta \right)=\frac{1}{s^{H}\left(\eta \right)R^{-1}\left(\eta \right)s\left(\eta \right)}\\ s\left(\eta \right)=s_{s}\left(\eta \right)⊗s_{t}\left(\eta \right)\\ s_{s}\left(\eta \right)={\left[1,e^{jw_{s}\left(\eta \right)}\right]}^{T}\\ s_{t}\left(\eta \right)={\left[1,e^{j1w_{t}\left(\eta \right)},e^{j2w_{t}\left(\eta \right)},\cdots ,e^{j(M-1)w_{t}\left(\eta \right)}\right]}^{T} \end{array}$$

μ(η) is a constant varied by periods. s(η)∈2M×1 represents the steering vector of the moving target and it can be obtained by the Kronecker product of the target’s space steering vector $s_{s}\left(\eta \right)\in 2\times 1$ and the time steering vector $s_{t}\left(\eta \right)\in M\times 1$. $w_{s}\left(\eta \right)$ and $w_{t}\left(\eta \right)$ are the space frequency and time frequency of the moving target at the period η, respectively. R(η)∈2M×2M is the clutter covariance matrix and $R^{-1}\left(\eta \right)\in 2M\times 2M$ is the inverse of it. 

In practice, due to the complex unknown environment, the covariance matrix R(η) need to be estimated from the training data, which is consisted by the echo from the l adjacent range cells. The estimation of R(η) is given by

(21) $$\hat{R}\left(\eta \right)=\frac{1}{l}\sum_{i=1}^{l}x_{i}\left(\eta \right)x_{i}^{H}\left(\eta \right)$$

The training data x(η) of the *i*-th range cell can be obtained by

(22) $$x\left(\eta \right)=vec\left\{{\left(rect\left(\frac{t_{1}-\eta -\left\lfloor \delta_{a}/\left(2\left|K_{a}\right|\right)\right\rfloor }{\left\lfloor \delta_{a}/\left|K_{a}\right|\right\rfloor }\right)\times \left[X_{1}(t_{1}),\cdots ,X_{k}(t_{1}),\cdots ,X_{N}(t_{1})\right]\right)}^{T}\right\}$$

where $X_{k}(\eta )\in M\times 1$ denotes the column vector of the sampling data received at k-th receive channel, ⌊⋅⌋ represents the operation whose result is a positive integer and its value is not greater than that in brackets.

**Figure 2:**
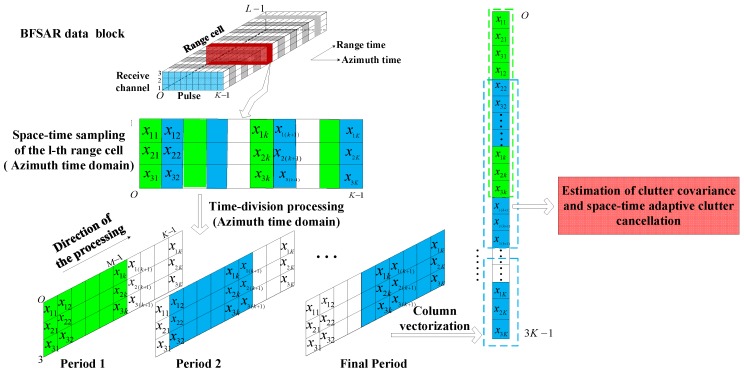
The procedure of first clutter cancellation in SAR echo domain.

We call this method time-division space-time adaptive clutter cancellation and its construction can be regarded as a sliding time-variant FIR filter, whose optimal weight vectors are updating during each period. The procedure of the first clutter cancellation in SAR echo domain is shown in Figure 2.

**Figure 3:**
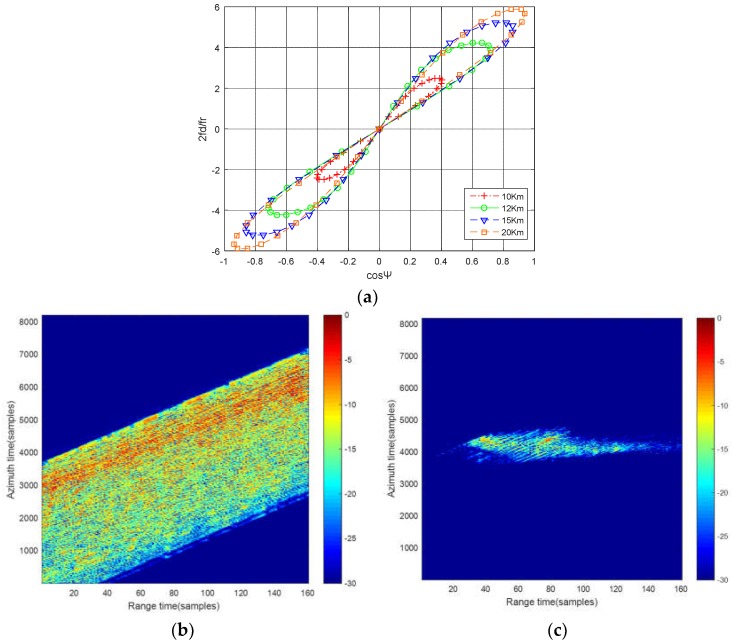
Time-division STACC process under close distance situation: (**a**) Clutter ridge of BFSAR; (**b**) The raw data before time-division STACC; (**c**) The result of two-channel time-division STACC.

With the time division processing, the first clutter cancellation can be achieved in SAR echo domain. However, the spectral distribution of the BFSAR clutter is closely related to the bistatic configuration, platform velocities and flight directions. That means the Doppler frequency of BFSAR clutter is range-dependent, namely BFSAR clutter is non-stationary, especially for the close distance range situations [32,33]. Due to the non-stationary characteristic of BFSAR clutter, the CCM can’t be estimated accurately. The result of the time-division STACC is badly deteriorated. The simulation result of the two-channel time-division STACC under close distance range situation is shown in Figure 3.

In Figure 3a, the lines in different colors present the space-time distribution of bistatic clutter with different bistatic ranges. As can be seen that the distribution of clutter varies with bistatic ranges under the close distance situation, namely the Doppler frequency of bistatic clutter is range-dependent. As a consequence, the samplings used for clutter plus noise covariance matrix estimation are not independent and identically distributed, which results in wrong weight calculation of the optimal filter. Furthermore, the performance of clutter suppression will be severely degraded.

Figure 3b is the raw data before time-division STACC and the signal to clutter and noise ratio (SCNR) is −11.34 dB. Figure 3c is the result of two-channel time-division STACC under the close distance situation. Compared with Figure 3b, it clearly shows that the range-dependent BFSAR clutter cannot be suppressed sufficiently and the SCNR in Figure 3c is −1.28 dB. The SCNR improvement is about 10 dB, but it is not enough for the moving target detection in the strong residual BFSAR clutter. Target signal is still buried in the clutter background. Target detection is difficult to achieve after the first clutter cancellation in echo domain.

### 3.4. Second Clutter Cancellation in Image Domain

In this section, we consider the problem of the residual non-stationary clutter cancellation. As mentioned above, the performance on clutter suppression will be heavily impacted due to the non-stationary characteristic of BFSAR clutter. Thus, ADPCA is introduced to deal with the second clutter cancellation in image domain. 

After the two-channel time-division STACC, the data of the channel $k_{1}$ is generated by the first time-division STACC and the data of the channel $k_{2}$ is generated by the second one. The target signal has been retained as much as possible according to the mathematical optimization model mentioned in Equation (18), thus the target signal of $k^{*}-th\;(k=k_{1},k_{2})$ channel in two-dimension time domain is given by

(23) $$S_{1}(t_{1},\tau ,k^{*})\approx I\left(\tau ,k^{*}\right)\omega_{a}\left(\frac{t_{1}-t_{R}}{T_{s}}\right)\exp\left\{-\frac{j2\pi f_{c}}{c}R_{a}(t_{1},k^{*})\right\}$$

where B is the bandwidth of the signal, and

(24) $$\begin{array}{l} I\left(\tau ,k^{*}\right)=\sin c\left[B\left(\tau -\frac{2R(0,k^{*};x,y)-R^{\prime \prime }(0,k^{*};x,y)t_{1}^{2}}{2c}\right)\right]\\ R_{a}(t_{1},k^{*})=R(0,k^{*})+(R^{\prime }(0,k^{*})-\lambda f_{dc})t_{1}+\frac{R^{\prime \prime }(0,k^{*})}{2}t_{1}^{2} \end{array}$$

The deramp function can be constructed as follows:

(25) $$C_{k1}\left(t_{1}\right)=\exp\left\{\frac{j2\pi }{\lambda }\left[\begin{array}{l} \left(-\left(v_{R}\cos\theta_{R,1}+v_{T}\cos\theta_{T,1}\right)-\lambda f_{dc}\right)t_{1}\\ +\left(\frac{V_{R}^{2}\sin^{2}\theta_{R,1}}{2R_{0R,1}}+\frac{V_{T}^{2}\sin^{2}\theta_{T,1}}{2R_{0T,1}}\right)t_{1}^{2} \end{array}\right]\right\}$$

(26) $$C_{k2}\left(t_{1}\right)=\exp\left\{\frac{j2\pi }{\lambda }\left[\begin{array}{l} \left(-\left(v_{R}\cos\theta_{R,2}+v_{T}\cos\theta_{T,2}\right)-\lambda f_{dc}\right)t_{1}\\ +\left(\frac{V_{R}^{2}\sin^{2}\theta_{R,2}}{2R_{0R,2}}+\frac{V_{T}^{2}\sin^{2}\theta_{T,2}}{2R_{0T,2}}\right)t_{1}^{2} \end{array}\right]\right\}$$

By multiplying with the deramp function mentioned above, we can have

(27) $$\begin{array}{l} S_{2}(t_{1},\tau ,k^{*})\approx I\left(\tau ,k^{*}\right)\omega_{a}\left(\frac{t_{1}-t_{R}}{T_{s}}\right)\exp{\left\{-\frac{j2\pi f_{c}}{c}\left(\begin{array}{l} R_{0R}(k^{*})+R_{0T}+\left(v_{rad,R}\left(k^{*}\right)+v_{rad,T}\right)t_{1}\\ +\left(A_{R}(k^{*})+A_{T}\right)t_{1}^{2} \end{array}\right)\right\}}_{2}\\ A_{T}=\frac{v_{x}^{2}-2v_{T}v_{y}+v_{y}^{2}}{2R_{0T}}-\frac{v_{x}^{2}{\left(x-x_{T}\right)}^{2}+(y-y_{T})(2v_{x}(x-x_{T})(v_{y}-v_{T})+v_{y}(v_{y}-2v_{T})(y-y_{T}))}{2R_{0T}^{3}}\\ \begin{array}{ll} A_{R}(k) & =\frac{v_{x}^{2}-2v_{R}v_{y}+v_{y}^{2}}{2R_{0R}}-\frac{v_{x}^{2}{\left(x-x_{R}\right)}^{2}+2v_{x}(y-y_{R}-(k-1)d)(x-x_{R})(v_{y}-v_{R})}{2R_{0R}^{3}}\\ & -\frac{v_{y}(v_{y}-2v_{R}){\left(y-y_{R}-(k-1)d\right)}^{2}}{2R_{0R}^{3}} \end{array} \end{array}$$

Due to $d\ll R_{0R}$, we can assume that $A_{R}=A_{R}(k_{1})\approx A_{R}(k_{2})$. And $A(v_{x},v_{y})=A_{R}+A_{T}$ is a function of the moving target velocity. In order to unify the imaging coordinate system, a time shift $\Delta t_{1}=\left(k_{2}-k_{1}\right)d/v_{R}$ is performed on the signal of the $k_{2}-th$ channel, then we can have

(28) $$\begin{array}{l} S_{3}(t_{1},\tau ,k_{1})\approx I^{\prime }\left(\tau \right)\exp\left\{-\frac{j2\pi }{\lambda }\left(R_{0R}(k_{1})+R_{0T}+\left(v_{rad,R}\left(k_{1}\right)+v_{rad,T}\right)t_{1}+A(v_{x},v_{y})t_{1}^{2}\right)\right\}\\ S_{3}(t_{1}+\Delta t_{1},\tau ,k_{2})\approx I^{\prime }\left(\tau \right)\exp\left\{-\frac{j2\pi }{\lambda }\left(\begin{array}{l} R_{0R}(k_{2})+R_{0T}+\left(v_{rad,R}\left(k_{2}\right)+v_{rad,T}\right)\left(t_{1}+\Delta t_{1}\right)\\ +A(v_{x},v_{y}){\left(t_{1}+\Delta t_{1}\right)}^{2} \end{array}\right)\right\}\\ I^{\prime }\left(\tau \right)=I\left(\tau ,k_{1}\right)\omega_{a}\left(\frac{t_{1}-t_{R}}{T_{s}}\right) \end{array}$$

Then, the coarse focused image of two channels can be obtained by taking the Fourier transform respect with $t_{1}$, and the image signals are given by

(29) $$\begin{array}{l} S_{4}(f_{t},\tau ,k_{1})\approx I^{\prime }\left(\tau \right)\exp\left\{-\frac{j2\pi }{\lambda }\left(R_{0R}(k_{1})+R_{0T}\right)\right\}F_{k1}(v_{x},v_{y})\\ S_{4}(f_{t},\tau ,k_{2})\approx I^{\prime }\left(\tau \right)F^{*}\exp\left\{-\frac{j2\pi }{\lambda }\left(R_{0R}(k_{2})+R_{0T}+\left(v_{rad,R}\left(k_{2}\right)+v_{rad,T}\right)\Delta t_{1}\right)\right\}F_{k2}(v_{x},v_{y}) \end{array}$$

where

(30) $$\begin{array}{l} F_{k1}(v_{x},v_{y})=\int_{0}^{T_{s}}\exp\left\{-j\frac{2\pi }{\lambda }\left((v_{rad,R}\left(k_{1}\right)+v_{rad,T}+\lambda f_{t})t_{1}+A(v_{x},v_{y})t_{1}^{2}\right)\right\}dt_{1}\\ F_{k2}(v_{x},v_{y})=\int_{0}^{T_{s}}\exp\left\{-j\frac{2\pi }{\lambda }\left(\begin{array}{l} (v_{rad,R}\left(k_{1}\right)+v_{rad,T}+\lambda f_{t}+2A(v_{x},v_{y})\Delta t_{1})t_{1}\\ +A(v_{x},v_{y})t_{1}^{2} \end{array}\right)\right\}dt_{1}\\ F^{*}=\exp\left\{-\frac{j2\pi }{\lambda }A(v_{x},v_{y})\Delta t_{1}{}^{2}\right\} \end{array}$$

In order to align the equivalent phase center of the different receive channels, the compensation function for the image of the $k_{2}-th$ channel is constructed as

(31) $$G=\exp\left\{-j\frac{2\pi }{\lambda }\left(R_{0R}(k_{1})-R_{0R}(k_{2})\right)\right\}$$

**Figure 4:**
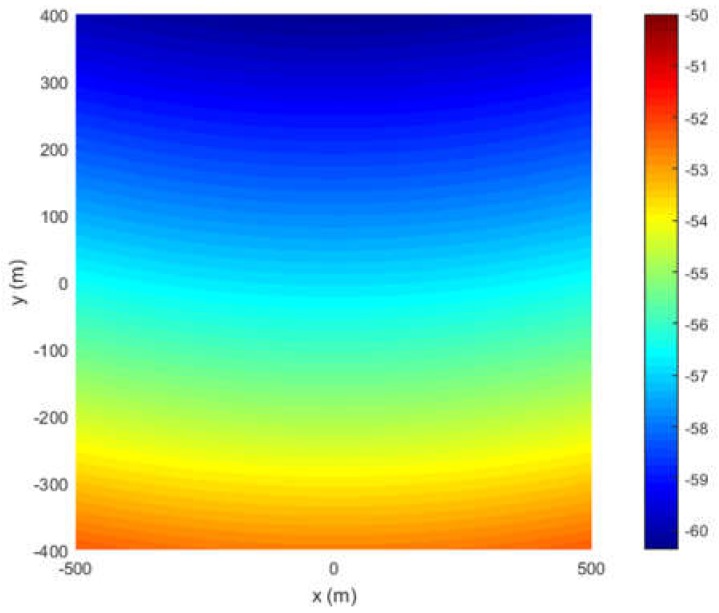
The spatial distribution of the phase in Equation (31).

The spatial distribution of the phase in Equation (31) is shown in Figure 4.

Finally, the second cancellation in image domain can be expressed as

(32) $$\Delta S(f_{t},\tau )=S_{4}(f_{t},\tau ,k_{1})-G\cdot S_{4}(f_{t},\tau ,k_{2})$$

For the residual clutter, we have

(33) $$\left\{ \begin{array}{l} v_{rad,R}\left(k\right)=0\\ v_{rad,T}=0\\ A(v_{x},v_{y})=0 \end{array}\right.\Rightarrow \{\begin{array}{c} F^{*}=1\\ F_{k1}(v_{x},v_{y})=F_{k2}(v_{x},v_{y}) \end{array}\Rightarrow \Delta S_{clutter}(f_{t},\tau )=0$$

For the ground moving target with the slow velocity, we have

(34) $$\begin{array}{ll} \{\begin{array}{c} F^{*}\approx 1\\ F_{k1}(v_{x},v_{y})\approx F_{k2}(v_{x},v_{y}) \end{array}\Rightarrow \Delta S_{t\arg et}(f_{t},\tau ) & =I^{\prime }\left(\tau \right)F_{k1}(v_{x},v_{y})\exp\left\{-j\frac{2\pi }{\lambda }\left(R_{0R}(k_{1})+R_{0T}\right)\right\}\\ & \times \left(1-\exp\left\{-j\frac{2\pi }{\lambda }\left(\left(v_{rad,R}\left(k_{2}\right)+v_{rad,T}\right)\right)\Delta t_{1}\right\}\right)\neq 0 \end{array}$$

As can be seen from Equations (33) and (34), after the second clutter cancellation in image domain, the residual non-stationary clutter can be cancelled sufficiently in the image domain and moving target is retained and coarse focused. The energy of moving target has been almost gathered in image. Then, the goal of the ground moving target detection for BFSAR can be achieved.

**Figure 5:**
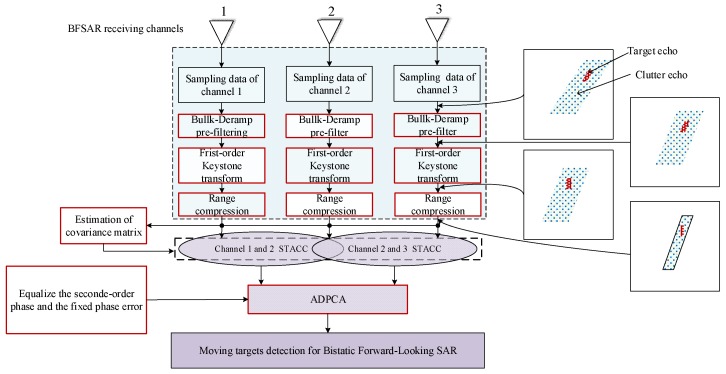
The procedure of the proposed method.

Heretofore, the ground moving target detection method for BFSAR with three receiving channels has been detailed. The procedure of the proposed method is given in Figure 5.

## 4. Experiments and Results

Experiments and results are given in this section to verify the effectiveness of the proposed method. In the experiments, the proposed method will be compared with the traditional STAP and direct three-channel time-division STACC method after Doppler ambiguity suppression and correction of cell migration. The performance on SCNR improvement and detection of these methods are analyzed. The simulation parameters are listed in Table 1.

The receiver is equipped with a 3-channel linear array along the flight direction, and the spacing d between channels is set to be 0.4 m. For the proposed method, the joint clutter cancellation is consisted of twice two-channel time-division STACC and adaptive DPCA. The first two-channel time-division STACC is conducted between channel 1 and 2, and the second one is between channel 2 and 3. And one processing period is set to be 15 pulses. For the ADPCA, the corresponding PRF is 2000 Hz [21,23]. 

**Figure 6:**
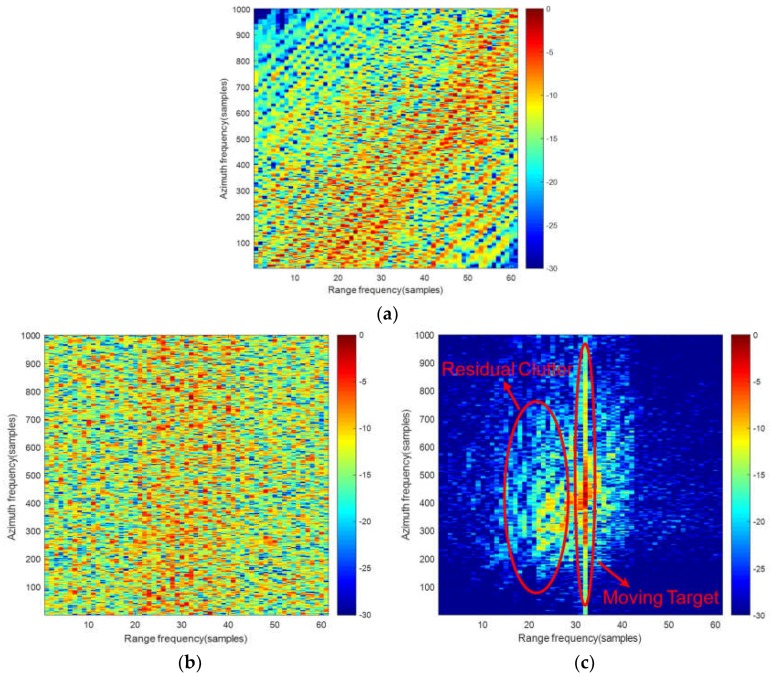
The results of traditional STAP and direct three-channel time-division STACC: (**a**) the echo data after keystone transform; (**b**) The result of traditional STAP; (**c**) The result of direct three-channel time-division STACC.

With the close detection range situation, namely the strong non-stationary ground clutter environment, we consider that the original coordinates of transmitter and receiver are (−3000,−5000,3000) m and (0,−3000,3000) m, respectively. Figure 6a shows the echo data after keystone transform. Figure 6b,c gives the results of traditional STAP and direct three-channel time-division STACC after first two steps. In Figure 6b, traditional STAP is conducted with Doppler ambiguity, range cell migration and Doppler cell migration. It can be seen that the performance of the traditional STAP is tremendously deteriorated and it’s impossible to detect a moving target from the non-stationary clutter background. Figure 6c is the result of the three-channel time-division STACC. It clearly shows that the non-stationary clutter cannot be suppressed sufficiently by the three-channel time-division STACC. A moving target is difficult to be distinguished with the clutter. Also, the false alarm will be caused by the residual clutter and the performance on detection is degraded.

**Figure 7:**
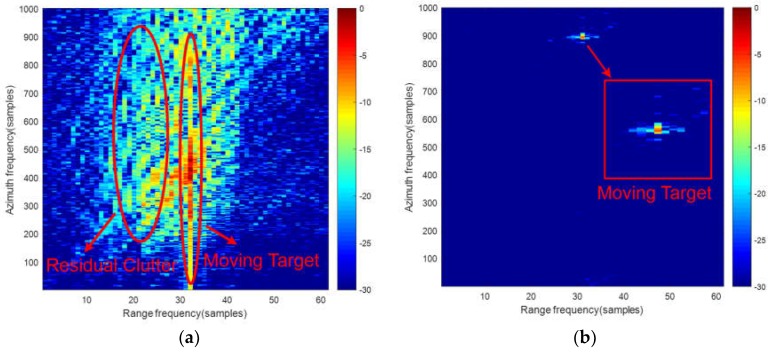
The result of joint clutter cancellation in echo-image domain: (**a**) the result of the first two-channel time-division STACC; (**b**) The result of ADPCA.

Figure 7 shows the result of joint clutter cancellation in echo-image domain. After the elimination of Doppler ambiguity and the correction of cell migration, the result of the first two-channel time-division STACC is shown in Figure 7a. Target signal is difficult to detect in Figure 7a. Figure 7b shows the result of ADPCA. It can be seen that only the moving target signal is retained and coarse focused. Thus, the purpose of moving target detection can be easily achieved. 

Table 2 shows the SCNR improvement of the three methods. From Table 2, it can be seen that the proposed method has better SCNR improvement than traditional STAP and direct three-channel time-division STACC. The SCNR can be improved 41.9234 dB by the proposed method. From the experimental results and SCNR improvement, it is clearly shown that the non-stationary clutter of BFSAR can be suppressed sufficiently by the proposed method and better detection performance can be achieved.

## 5. Conclusions

In this paper, an effective BFSAR ground moving target detection method based on joint clutter cancellation with three receiving channels is proposed. Considering the Doppler ambiguity and cell migration of BFSAR, the proposed method mainly concludes four steps. First, the bulk-deramp pre-filtering is applied to suppress the Doppler ambiguity. Then, the first-order keystone transform is the second step to correct the large-scale range cell migration. The energy of the moving target will be gathered into one range cell. After the steps mentioned above, two-channel time-division STACC will be conducted between two adjacent channels as the third steps to suppress the BFSAR clutter at the first time in echo domain without the effect of the migration of Doppler cells. Due to the bad performance of the non-stationary BFSAR clutter suppression, ADPCA is exploited as the fourth step to cancel the residual non-stationary clutter in image domain. Experimental results have shown that the non-stationary clutter of BFSAR can be suppressed sufficiently after the joint clutter cancellation and the SCNR improvement of the propose method is greater than other methods. The GMTD performance of the proposed method makes it easy to achieve the goal of ground moving target detection.

## Figures and Tables

**Table 1:** Simulation parameters.

Parameters	Value
Center Frequency	9.6 GHz
Range Bandwidth	100 MHz
Synthetic Aperture Time	0.5 s
Transmitter’s Velocity	(0,200,0) m/s
Receiver’s Velocity	(0,200,0) m/s
Target Center	(0,0,0) m
Target Velocity	(3,−3,0) m/s

**Table 2:** SCNR Improvement of three methods.

	SNCR	SCNR Improvement
After keystone transform	−9.9019	/
Traditional STAP	−3.6283	6.2736 dB
Direct STACC	14.1097	23.2016 dB
Joint clutter cancellation	32.0215	41.9234 dB
